# MR CLEAN, a multicenter randomized clinical trial of endovascular treatment for acute ischemic stroke in the Netherlands: study protocol for a randomized controlled trial

**DOI:** 10.1186/1745-6215-15-343

**Published:** 2014-09-01

**Authors:** Puck SS Fransen, Debbie Beumer, Olvert A Berkhemer, Lucie A van den Berg, Hester Lingsma, Aad van der Lugt, Wim H van Zwam, Robert J van Oostenbrugge, Yvo BWEM Roos, Charles B Majoie, Diederik WJ Dippel, for the MR CLEAN Investigators

**Affiliations:** Department of Neurology, Erasmus MC University Medical Center, PO Box 2040, 3000 CA Rotterdam, the Netherlands; Department of Radiology, Erasmus MC University Medical Center, PO Box 2040, 3000 CA Rotterdam, the Netherlands; Department of Neurology, Maastricht University Medical Centre, PO Box 5800, 6202 AZ Maastricht, the Netherlands; Department of Radiology, Academisch Medisch Centrum, PO Box 22660, 1100 DD Amsterdam, the Netherlands; Department of Neurology, Academisch Medisch Centrum, PO Box 22660, 1100 DD Amsterdam, the Netherlands; Department of Public Health, Erasmus MC University Medical Center, PO Box 2040, 3000 CA Rotterdam, the Netherlands; Department of Radiology, Maastricht University Medical Centre, PO Box 5800, 6202 AZ Maastricht, the Netherlands

**Keywords:** Alteplase, Urokinase, Endovascular treatment, Acute ischemic stroke, Randomized controlled trial, Stent, Thrombectomy

## Abstract

**Background:**

Endovascular or intra-arterial treatment (IAT) increases the likelihood of recanalization in patients with acute ischemic stroke caused by a proximal intracranial arterial occlusion. However, a beneficial effect of IAT on functional recovery in patients with acute ischemic stroke remains unproven. The aim of this study is to assess the effect of IAT on functional outcome in patients with acute ischemic stroke. Additionally, we aim to assess the safety of IAT, and the effect on recanalization of different mechanical treatment modalities.

**Methods/design:**

A multicenter randomized clinical trial with blinded outcome assessment. The active comparison is IAT versus no IAT. IAT may consist of intra-arterial thrombolysis with alteplase or urokinase, mechanical treatment or both. Mechanical treatment refers to retraction, aspiration, sonolysis, or use of a retrievable stent (stent-retriever). Patients with a relevant intracranial proximal arterial occlusion of the anterior circulation, who can be treated within 6 hours after stroke onset, are eligible. Treatment effect will be estimated with ordinal logistic regression (shift analysis); 500 patients will be included in the trial for a power of 80% to detect a shift leading to a decrease in dependency in 10% of treated patients. The primary outcome is the score on the modified Rankin scale at 90 days. Secondary outcomes are the National Institutes of Health stroke scale score at 24 hours, vessel patency at 24 hours, infarct size on day 5, and the occurrence of major bleeding during the first 5 days.

**Discussion:**

If IAT leads to a 10% absolute reduction in poor outcome after stroke, careful implementation of the intervention could save approximately 1% of all new stroke cases from death or disability annually.

**Trial registration:**

NTR1804 (7 May 2009)/ISRCTN10888758 (24 July 2012).

**Electronic supplementary material:**

The online version of this article (doi:10.1186/1745-6215-15-343) contains supplementary material, which is available to authorized users.

## Background

### Intravenous thrombolysis

Treatment with intravenous (IV) alteplase, aiming at early reperfusion, has been proven effective for patients with acute ischemic stroke when they are treated within 4.5 hours after stroke onset. The number of patients eligible for treatment with IV alteplase is limited because of the restricted time window [1–3]. In approximately 33% of the patients with acute anterior circulation ischemic stroke, symptoms are caused by a proximal occlusion of one of the major intracranial arteries - that is, the distal intracranial carotid artery, the proximal segment of the middle cerebral artery and the anterior cerebral artery [4]. The likelihood of a proximal occlusion increases with severity of neurological deficit at presentation [5, 6]. In these patients the effect of IV alteplase is limited and leads to recanalization in only 33% of the cases. In patients without recanalization outcome is generally poor [7].

### Intra-arterial treatment

Delivery of the thrombolytic agent at the site of the occlusion may improve the likelihood of recanalization, reperfusion of still viable tissue and, hence, recovery of neurological deficits. Several randomized clinical trials of intra-arterial treatment (IAT) for acute ischemic stroke have been conducted and published [8–10]. Although the results of these trials suggested a benefit, they have to be interpreted with care and cannot be extrapolated to the current clinical situation since IV alteplase was not an option, neither as pre-treatment nor as part of the control treatment. In the Middle cerebral artery Embolism Local fibrinolytic intervention Trial, mechanical treatment was allowed [10], but this was not available in Prolyse in Acute Cerebral Thromboembolism (PROACT) I or PROACT II [8, 9].

The Interventional Management of Stroke III (IMS-III) trial was an international randomized, multicenter, open label trial of the effect of combined IV/IAT versus IV treatment only, when treatment is initiated within 3 hours in patients with a National Institutes of Health stroke scale (NIHSS) score ≥10. The sponsor terminated the trial prematurely because of futility; there were no safety concerns. The IMS-III included 656 patients who were all treated intravenously. The increase in absolute risk of good outcome (modified Rankin Scale (mRS) ≤2) at 3 months follow-up was 1.5% (95% CI -6.1 to 9.1%). Several factors may have contributed to the absence of a treatment effect: confirmation of occlusion was not required at the time of randomization; in the IV/IAT group, 23% of the patients did not receive IAT due to the absence of an arterial occlusion; time from onset of symptoms to IAT was rather long (249 minutes on average); and only five patients (1.2%) were treated with a stent-retriever [11].

SYNTHESIS Expansion was a head-to-head comparison of IAT with IV treatment in 362 patients. In the intervention group, 4.4% fewer patients recovered (95% CI -14.6 to 5.8%) than in the group with standard treatment. Time from onset of symptoms to treatment was on average 225 minutes in the IAT group, but patients receiving the standard treatment with IV recombinant tissue Plasminogen Activator (rtPA) were treated 60 minutes earlier. In this study, confirmation of an occlusion at the time of randomization was not required and only a small group of patients was treated with a stent-retriever (23 patients, 12.7%) [12].

### Intravenous and intra-arterial thrombolytic treatment

The combination of IV and intra-arterial alteplase has been described in observational studies and in one other randomized controlled trial [13]. Some studies adjusted the intravenous dose to 0.6 mg/kg, with a maximum dose of 60 mg. The incidence of hemorrhages was no larger than in studies of treatment with IV thrombolysis only [14–18]. In case series, IAT with low dose intra-arterial alteplase was preceded by full dose IV alteplase (that is, 0.9 mg/kg). Risk of symptomatic intracerebral hemorrhage ranged from 0 to 13% [19–21]. These studies suggest that, in patients who have been treated this way, recanalization rates can be high without unacceptably high risks of complications.

### Mechanical thrombectomy

The Mechanical Retrieval and Recanalization of Stroke Clots Using Embolectomy trial compared mechanical thrombectomy with the MERCI Retriever (Concentric Medical, Mountain View, USA) with standard therapy in 118 patients who had undergone IV thrombolysis. All patients underwent computed tomography (CT) perfusion or magnetic resonance imaging (MRI) diffusion/perfusion, and randomization was stratified for the presence of penumbra. This study showed no beneficial effect of the intervention overall, or in the pre-stratified subgroup of patients with penumbra [22].

Since the first application of IAT for acute ischemic stroke, new techniques for mechanical treatment have been developed. Mechanical treatment is a promising approach, either as a primary intervention or as secondary treatment in patients who fail IV thrombolysis, or in patients for whom thrombolytic agents are contraindicated. Mechanical techniques include retraction, aspiration, stenting and other techniques, such as local ultrasound-augmented fibrinolysis. Studies suggest that, in experienced hands, mechanical thrombectomy devices can be safe and may lead to substantial recanalization rates [23]. The results of two randomized clinical trials comparing retrievable stents with a retraction device suggest that use of a retrievable stent leads to recanalization more often than use of a retraction device. No comparison was made with standard treatment [24, 25].

### Research question

The MR CLEAN aims to assess the effect of IAT on functional outcome in patients with acute ischemic stroke caused by a proximal intracranial arterial occlusion.

## Methods/design

### Design

**Figure 1:**
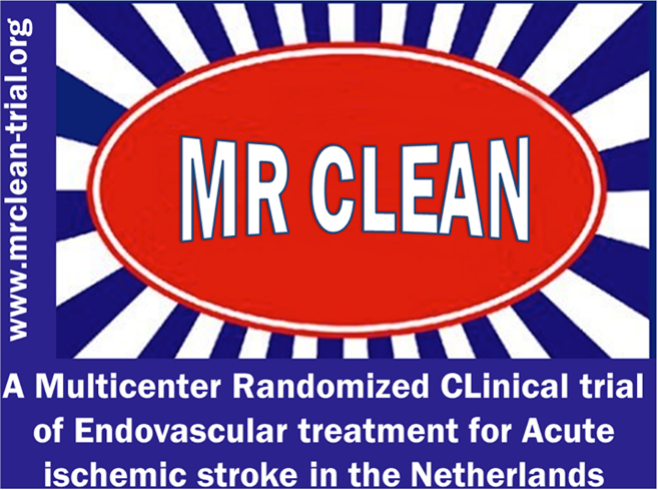
**Trial logo.**

The Multicenter Randomized Clinical trial of Endovascular treatment for Acute ischemic stroke in the Netherlands (MR CLEAN) is a multicenter clinical trial with randomized treatment allocation, open-label treatment and blinded endpoint evaluation (PROBE design) (Figure 1). The active comparison is IAT (intra-arterial alteplase or urokinase, and/or mechanical treatment) versus no IAT. The treatment is provided in addition to best medical management according to national and international guidelines, and may include IV thrombolysis. The study currently runs in 17 large hospitals in the Netherlands for a total period of 5 years (4 years of patient inclusion). Patient inclusion started in December 2010.

### Patient inclusion and exclusion criteria

Patients aged 18 years or older with acute ischemic stroke and a symptomatic anterior proximal artery occlusion, which can be treated within 6 hours after stroke onset, are eligible for participation in this trial.

General inclusion criteria are: a clinical diagnosis of acute stroke with a deficit on the NIHSS of at least 2 points, CT or MRI ruling out intracranial hemorrhage, occlusion of distal intracranial carotid artery or middle (M1 or M2 or anterior cerebral artery (A1) demonstrated with CT angiography (CTA), magnetic resonance angiography (MRA) or digital subtraction angiography (DSA), the possibility to start treatment within 6 hours of onset, aged 18 years or over and informed consent given in writing.

We use three sets of exclusion criteria: general exclusion criteria for IAT, a specific exclusion criterion for mechanical treatment and specific exclusion criteria for intra-arterial thrombolysis.

General exclusion criteria are: arterial blood pressure exceeding 185/110 mmHg, blood glucose less than 2.7 or over 22.2 mmol/L, treatment with IV thrombolysis in a dose exceeding 0.9 mg/kg or 90 mg or treatment with IV thrombolysis despite contraindications, and, finally, cerebral infarction in the distribution of the relevant occluded artery in the previous 6 weeks.

A specific exclusion criterion for intended mechanical thrombectomy is laboratory evidence of coagulation abnormalities (that is, platelet count <40 × 10^9^/L, activated partial Thromboplastin time (APTT) >50 seconds or International normalized ratio (INR) >3.0).

Specific exclusion criteria for intended intra-arterial thrombolysis are: a history of cerebral hemorrhage, severe head injury (contusion) in the previous 4 weeks and clinical laboratory evidence of coagulation abnormalities, (that is, platelet count <90 × 10^9^/L, APTT >50 seconds or INR >1.7), or treatment with oral thrombin or factor X antagonists.

These hierarchically ordered exclusion criteria make it possible that patients with contraindications for IV or IAT with alteplase but no contraindication for mechanical thrombectomy are included in the study. Also, patients who cannot be treated within the 4.5-hour time window may still be included in the trial. Enrolment was not limited according to the Alberta Stroke Program Early CT Score (ASPECTS) or extension of early signs of infarction at baseline.

### Eligibility criteria for participating centers

To be fully eligible for participation in the trial and to include patients in the trial, centers should meet the following minimum criteria. The intervention team should have ample experience with intra-arterial interventions for cerebrovascular disease (carotid stenting or aneurysm coiling), peripheral artery disease, or coronary artery disease. In order to include and randomize patients who may be treated with mechanical thrombectomy, the intervention team should make use of one or more of the devices that have been approved by the trial steering committee. Other devices are not allowed into the trial. At least one member of the intervention team should have sufficient experience with IAT for acute ischemic stroke and with the particular device that is being used (sufficient experience in this context is defined as the completion of at least five procedures with the particular device or procedure). Compliance with these criteria is checked by the data-monitor.

### Randomization

The randomization procedure is computer- and web-based, using permuted blocks. Full-time back-up by telephone is provided. Randomization is allowed when the intracranial occlusion has been established by CTA, MRA or DSA. Randomization is stratified for center, use of IV alteplase, planned treatment modality (mechanical thrombectomy or not) and stroke severity (NIHSS >14 or not).

### Intervention

IAT will consist of arterial catheterization with a microcatheter to the level of occlusion and delivery of a thrombolytic agent and/or mechanical thrombectomy. The decision for intubation or conscious sedation was left to the treating physicians. Time from onset to treatment (needle in groin) was recorded. The trial steering committee has issued recommendations that interventional procedures should be stopped at 8 hours from onset of symptoms.

Both alteplase and urokinase for intra-arterial thrombolysis are allowed into the trial, a dose of 1 mg alteplase is considered to be equivalent to 10,000-15,000 U urokinase. Patients who are pre-treated with IV alteplase should not receive more than 30 mg alteplase or 400,000 U urokinase intra-arterially. The maximum allowed dose of urokinase is 1,200,000 U urokinase [26]. Mechanical treatment may consist of thrombus retraction, aspiration, sonolysis or use of a retrievable stent. Specific recommendations with regards to procedures and devices will be issued regularly by the trial steering committee.

The steering committee will make recommendations for dosages of thrombolytic agents, procedures, and for devices that will be allowed in the trial based on proposals by the executive committee or local investigators. The requirements for a device to be allowed in the trial are Conformité Européenne (CE) marking or Food and Drug Administration (FDA) registration, documented evidence of safety in experienced hands, recanalization rates that are similar to rates with other mechanical devices, and published case series of at least 20 patients with one particular type of device in a representative series of patients.

### Blinding

Both patient and treating physician will be aware of the treatment assignment. Treatment assignment cannot be determined before inclusion and randomization. Information on outcome at 3 months will be assessed with standardized forms and procedures, in a structured telephone interview by an experienced research nurse at the central trial office who is not aware of treatment allocation. Assessment of outcome on the mRS will be based on this information, by assessors who are blind to the allocated and actually received treatment. Results of neuroimaging will also be assessed by blinded observers. Information on treatment allocation and primary outcome will be kept separate from the main study database. The steering committee will be kept unaware of the results of interim analyses of outcomes, efficacy and safety. The trial statistician (HL) will combine data on treatment allocation and outcomes in order to report to the data monitoring committee (DMC).

### Study outcomes

The primary outcome is the score on the mRS at 90 days. Secondary outcomes are the imaging parameter vessel recanalization at 24 hours (Clot Burden score and collateral score), infarct size at 5 days assessed with ASPECTS, and final infarct volume calculation [27]. For clinical outcome, the NIHSS and NIH supplemental motor scale [28] at 24 hours and at 1 week or discharge will be assessed. To further assess functional outcome at 90 days, the score on the EuroQol 5D measurement tool for health-related quality of life and Barthel index will be used (Table 1) [29, 30]. DSA runs are evaluated by a separate independent central core laboratory because the assessors of DSA will not be blinded to treatment allocation.

**Table 1:** **Clinical assessment and neuroimaging at baseline and follow-up**

Baseline	
Clinical assessment	Demographics, risk factors, medication, medical history, NIHSS
Neuro-imaging	Unenhanced CT and CT angiography*
**Follow-up**	
Clinical assessment at 24 hours	NIHSS. Adverse events.
Neuro-imaging at 24 hours	CT angiography*
Neuro-imaging at 5-7 days	Unenhanced CT or MRI
Clinical assessment at 1 week or discharge	NIHSS; Barthel index
Clinical assessment at 90 days	Modified Rankin score, Barthel index, EQ5D

*Magnetic resonance imaging (MRI) and magnetic resonance angiography (MRA) are allowed; computed tomography (CT) perfusion is recommended but not obligatory. EQ-5D, EuroQol 5D measurement tool for health-related quality of life; NIHSS, National Institutes of Health stroke scale.

### Safety aspects

Safety is an issue of concern, as experience with the intervention overall and within the participating centers is limited. Adverse events are undesirable experiences occurring to subjects during the study, whether or not they are considered to be related to the experimental treatment. All adverse events reported spontaneously by the subject or observed by the investigators are recorded. A serious adverse event is defined as any untoward occurrence or effect that causes death, is life-threatening, requires prolonged hospitalization or results in persistent significant disability. The primary safety parameter will be neurologic deterioration during the first 24 hours from inclusion. This is defined as an increase in NIHSS of 4 points or more. Expected serious adverse events are neurologic deterioration, symptomatic intracranial hemorrhage, extracranial hemorrhage, technical complications or vascular damage at the target lesion, such as perforation or dissection, distal emboli in non-involved arteries, aspiration pneumonia, and allergic reactions to contrast agents.

### Data monitoring committee

The DMC is chaired by a neurologist, and includes a neuro-interventionist and a statistician (see Appendix 1). The DMC meets at least annually, and is provided with structured unmasked reports, prepared by the trial statistician, for their eyes only. The DMC assesses the occurrence of unwanted effects by center and by allocated treatment. During the period of intake to the study, interim analyses of mortality and of any other information that is available on major endpoints (including serious adverse events believed to be due to treatment) will be supplied, in strict confidence, to the chairman of the DMC along with any other analyses that the Committee may request. In the light of these analyses, the DMC will advise the chairman of the Steering Committee if, in their view, the randomized comparisons in MR CLEAN have provided both (1) "proof beyond reasonable doubt" that for all, or for some specific types of patients, one particular treatment is clearly indicated or clearly contraindicated in terms of a net difference in outcome, and (2) evidence that might reasonably be expected to materially influence patient management. Appropriate criteria of proof beyond reasonable doubt cannot be specified precisely, but a difference of at least 3 standard deviations in an interim analysis of a major endpoint may be needed to justify halting, or modifying, the study prematurely. This criterion has the practical advantage that the number of interim analyses is of little importance.

There are no detailed safety stopping rules. Safety criteria for individual centers include the following. If the local investigator or other member of the team at a trial center has a concern about the outcome of their trial procedures, they should inform the MR CLEAN trial office, which will organize a blinded assessment of the relevant outcome events. This will be submitted by the central office to the chairman of the DMC, who may recommend further action, such as suspending randomization at the center. Similarly, the database manager at the trial office will monitor outcome events and if there are three consecutive deaths or three consecutive serious adverse events at a single center within 30 days of treatment in the same arm of the study, then assessment of the events will be triggered. A cumulative death rate of more than 50% or a cumulative serious adverse event rate exceeding 20% over 10 cases during hospital admission would also trigger careful assessment of the relevant outcome events.

### Statistical analyses

Baseline characteristics will be summarized by means of simple descriptive statistics. The main analysis of this trial consists of a comparison of the primary outcome after 90 days between the trial treatment groups. The analysis will be based on the intention-to-treat principle. The primary effect parameter takes the whole range of the mRS into account and is defined as the relative risk for improvement on the mRS. It is estimated as an odds ratio with ordinal logistic regression [31, 32]. In this primary analysis, multivariable regression analysis will be used to adjust for chance imbalances in main prognostic variables between intervention and control group, such as age, stroke severity (NIHSS), time since onset, previous stroke, atrial fibrillation, carotid top occlusion and diabetes mellitus. Accordingly, treatment effect modification will be explored in subgroups defined by (tertiles of) these prognostic variables.

Secondary effect parameters will be the improvement according to the classic dichotomizations of the mRS scale at 0-1 versus 2-6 and 0-2 versus 3-6, vessel patency on CTA, MRA or DSA at 24 hours, and the score on the NIHSS at 24 hours and 1 week or discharge.

For the analysis of the secondary outcomes, simple 2 × 2 tables, two-group *t*-tests, Mann–Whitney tests, and multivariable linear and logistic regression models will be used, where appropriate. In all analyses, statistical uncertainty will be expressed by means of 95% CI. A detailed statistical analysis plan can be found in Additional file 1.

### Sample size

A moderate effect on the distribution of mRS scores, resulting in a 10% absolute increase in the cumulative proportion of patients with mRS 0-3 in the intervention group is assumed, compared with controls. The distribution of outcome categories is based on the results of the PROACT-II trial [9]. A total study size of 500 patients (2 × 250 patients) allows for a power (1-abeta) of 82% at a significance level of 0.05, taking into account 10% cross-over rate [33]. This sample size should also be sufficient to assess the effect of the intervention on secondary endpoints: analysis of a meaningful reduction on NIHSS at 1 week of 3-4 points (Cohen’s d = 0.33) would require a sample of 400 patients, assuming that at 24 to 48 hours mean NIHSS would be 12, with a standard deviation of 10. A doubling of the recanalization rate from 30% to 60% would require 126 patients to achieve a power of 0.90.

### Study organization and funding

See Appendix 1 for the study investigators. The trial steering committee is the main decision-making body. It consists of local principal investigators, a stroke neurologist and a neuro-interventionist from each participating center, the members of the executive committee, and the trial statistician. The steering committee meets at least once a year. The trial executive committee consists of a team of six principal investigators, the three coordinating junior researchers and the trial statistician. The trial executive committee also forms the writing committee for the trial. Publications will be made on behalf of all investigators.

All incoming data are reviewed by the trial coordinator at the central trial office. Imaging data are reviewed at the secondary imaging center. All data were entered into a web-based trial management system that allowed for edit and audit trails, by trained local research nurses. All local data were carefully reviewed and first three, as well as every 10th patient case report form was fully checked against source data. Subcommittees exist for outcome assessment, adverse event adjudication and imaging assessment.

### Ethical considerations

Informed consent will be obtained from all participants or their legal representative, in writing, before inclusion in the trial. The MR CLEAN trial protocol has been approved for the Netherlands by the central medical ethics committee and research board of Erasmus MC University Medical Center (MEC-2010-041).

## Discussion

MR CLEAN is a pragmatic multicenter randomized clinical trial of IAT for acute ischemic stroke versus no IAT. The study is a pragmatic phase III trial with a PROBE design. This trial will primarily evaluate the effect of IAT on functional outcome; secondarily it will assess the safety of IAT, and recanalization rates. MR CLEAN also aims to collect data for a cost-effectiveness evaluation. Furthermore, this trial will provide a basis for the further implementation of IAT in the Netherlands and other countries.

For the trial results to be generalizable and representative of the state-of-the-art approach in IAT, the trial design is pragmatic. This implies the possibility to use several local thrombolytic agents and mechanical devices for a broad range of patients with acute ischemic stroke caused by a proximal thrombo-embolic occlusion of one of the intracranial arteries belonging to the anterior circulation.

The trial’s pragmatism is also apparent from the clinical situations it addresses: patients who have been treated unsuccessfully with IV thrombolysis, patients who can be treated within 6 hours, but do not meet the time window requirements for IV thrombolysis, and patients with contraindications for IV or intra-arterial thrombolytic treatment (thrombectomy only).

This trial’s design is based on the existence of clinical equipoise, meaning that there is genuine uncertainty in the expert medical community over whether IAT will be beneficial [34]. The trial design accommodates the grey area of uncertainty principle; we allow different ranges of uncertainty with regard to eligibility related to age, stroke severity and other clinical or radiological criteria. We presume that all grey areas eventually overlap and will allow us to analyze the full clinical spectrum of acute ischemic stroke caused by intra-arterial occlusion [35].

### Limitations and concerns

We estimated a sample size of 500 patients. Although the sample size seems rather small, especially for a phase III intervention trial, it allows us to estimate the primary effect parameter with sufficient precision. Indeed, we made a quite conservative estimate of the treatment effect (10% absolute reduction in death and dependence), which is similar in size to the average effect of IV alteplase.

We do not restrict the use of multiple IAT modalities per patient. This is a limitation of the trial design, because it will restrict the possibilities of comparing different treatment modalities and only allows us to give a global judgment whether or not IAT is effective. On the other hand, this pragmatism allows us to follow current practice closely, and allows new mechanical devices and treatment strategies into the trial, even after the start of the study.

A concern with phase III trials of new interventions is the possibility of a “learning curve” - that is, an increase in effectiveness or decrease in the occurrence of procedure-related complications during the conduct of the trial. We therefore required a certain amount of experience with intra-arterial interventions from each group of devices and the number of procedures done by the interventionist. Moreover, we carefully gathered information on consecutive patients treated in each center before the start of the trial, in order to document the experience with the procedures. These data will be used to test for the presence of a learning curve in the trial data and before the start of the trial. Also, all participating centers register consecutive patients with acute ischemic stroke and record IATs given outside the trial protocol. These will be reported [36].

Time since onset is a serious concern. The arguments for a 6-, or even 8-hour time window from onset of stroke symptoms to treatment are mostly based on tradition (previous studies of IAT also used this window), and the absence of an association of complications and treatment effect with time since onset in previous, mostly neutral trials. However, we consider it likely that a treatment effect, if present, will be stronger in patients who can be treated early after onset of symptoms and we will encourage our investigators to act accordingly.

The primary effect parameter is defined as the relative risk for improvement on the mRS estimated as an odds ratio with ordinal logistic regression. The method is also called “shift analysis”, as it takes changes along the full range of the modified Rankin scale into account [31, 32]. It is therefore more sensitive to differences in outcome between the intervention and control groups, and also more relevant, as improvements will be taken into consideration that would not be registered as such in an analysis of dichotomized outcomes.

We assess recanalization on CTA because it is available in intervention and control patients. We will use a combination of Clot Burden Score and collateral flow score to assess the presence and extent of recanalization, because Thrombolysis in Myocardial Infarction (TIMI) or Thrombolysis in Cerebral Infarction (TICI) scores do not apply, as they require information concerning flow, which is not provided by CTA. We do not repeat DSA at 24 hours, as this would pose an additional risk to the patients in the study.

MR CLEAN has started in the Netherlands. We see no problems in generalizing our results to other countries in Western Europe and beyond. We strive to collaborate with other investigators and combine our data in a systematic review for effect estimates and prognostic modeling.

### Other ongoing trials

Several randomized clinical trials of intra-arterial therapy for acute ischemic stroke are ongoing. One trial exclusively concerns the treatment of patients with basilar artery occlusion [37]. Several other studies compare mechanical thrombectomy with standard treatment, both including, or preceded by IV alteplase [37–42]. Several other trials include patients who are ineligible for IV alteplase treatment exclusively [43] or additionally [44, 45]. Several trials have an upper age limit [39, 38, 42, 43, 45, 46], some exclude patients with a large ischemic core [44–46], and some require perfusion mismatch on baseline imaging [41, 45, 46].

Compared to these ongoing trials, MR CLEAN has the advantage of a short time window for inclusion and treatment and no restrictions in age, stroke severity or in penumbral imaging, all of which have not been validated sufficiently in our view. Moreover, the intervention is not restricted to one type or make of mechanical device.

### Expected benefit

In the Netherlands more than 44,000 patients suffer from stroke each year, 80% of these concern ischemic stroke. About a third of these patients arrive within 6 hours in a hospital. Of these, we expect about 33% to have a proximal intracranial occlusion [4]. A positive trial result could lead to at least a 10% absolute reduction in poor outcome. This implies that after successful implementation of the treatment in routine practice, almost 10% of all stroke patients could be treated and benefit. For the Netherlands, more than 400 patients would thus be saved from death or a disabled life, but for Europe as a whole this could amount to more than 10,000 patients annually [47].

We expect that MR CLEAN will increase our knowledge of the effects of IAT for acute ischemic stroke, and facilitate the further development and implementation of this potentially beneficial treatment.

## Trial status

**Figure 2:**
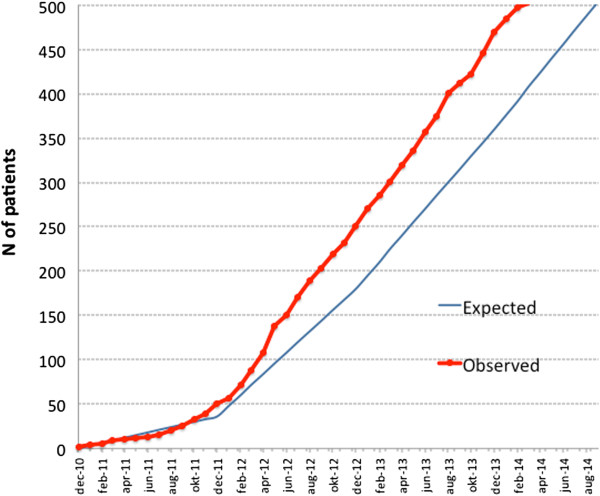
**Observed and expected accrual.** As of 16 March 2014, 500 patients were included in the trial.

Patient recruitment started in December 2010. Inclusion was completed with 500 patients on 16 March 2014 (Figure 2).

## Appendix 1: The MR CLEAN investigators

### Local principal investigators

Diederik W Dippel, Patrick A Brouwer, Erasmus MC Rotterdam; Yvo B Roos, Charles B Majoie, Academisch Medisch Centrum Amsterdam; Robert J van Oostenbrugge, Wim H van Zwam, Maastricht UMC. Jelis Boiten, Geert J Lycklama à Nijeholt, MC Haaglanden Den Haag; Marieke J Wermer, Marianne A van Walderveen, Leids Universitair Medisch Centrum Leiden; L Jaap Kappelle, Rob T Lo, UMC Utrecht; Ewoud J van Dijk, Joost de Vries, UMC St Radboud Nijmegen; Wouter J Schonewille, Jan Albert Vos, St Antonius Ziekenhuis Nieuwegein; Jeannette Hofmeijer, Jacques A van Oostayen, Rijnstate Ziekenhuis Arnhem; Patrick C Vroomen, Omid Eshghi, UMC Groningen; Paul L de Kort, Willem Jan van Rooij, St Elisabeth Ziekenhuis Tilburg; Koos Keizer, Xander Tielbeek, Catharina Ziekenhuis Eindhoven; Bas F de Bruijn, Lukas C van Dijk, Haga Ziekenhuis Den Haag; JS Peter van den Bergh, Boudewijn A van Hasselt, Isala Klinieken, Zwolle; Leo A Aerden, René J Dallinga, Reinier de Graaf Gasthuis, Delft; Tobien H Schreuder, Roel J Heijboer, Atrium MC Heerlen; Heleen M den Hertog, Dick G Gerrits, Medisch Spectrum Twente Enschede; Marieke C Visser, Joost C Bot, VUMC Amsterdam.

### Executive committee

Diederik WJ Dippel, Erasmus MC Rotterdam; Aad van der Lugt, Erasmus MC Rotterdam; Charles B Majoie, AMC Amsterdam; Yvo BWEM Roos, AMC Amsterdam; Robert J van Oostenbrugge, Maastricht UMC; Wim H van Zwam, Maastricht UMC.

### Imaging assessment committee

Charles B Majoie, chair; Wim H van Zwam; Geert J Lycklama à Nijeholt; Marianne A van Walderveen, Joost C Bot; Henk A Marquering; Ludo F Beenen; Marieke E Sprengers; Sjoerd Jenniskens, René van den Berg; Aad van der Lugt.

Independent DSA reader: Albert J Yoo, Massachussets General Hospital, Boston, USA.

### Outcome assessment committee

Yvo B Roos, chair; Peter J Koudstaal; Jelis Boiten; Ewoud J van Dijk.

### Adverse event committee

Robert J van Oostenbrugge, chair; Marieke J Wermer; H Zwenneke Flach.

### PhD-students and study coordinators

Puck SS Fransen, Erasmus MC Rotterdam; Debbie Beumer, UMC Maastricht; Olvert A Berkhemer, AMC Amsterdam, Lucie van den Berg, AMC Amsterdam.

### Trial statisticians

Ewout W Steyerberg, Dept of Public Health, Center for Clinical Decision Sciences, Erasmus MC Rotterdam. Hester F Lingsma, Dept of Public Health, Center for Clinical Decision Sciences Erasmus MC Rotterdam.

### Data Monitoring Committee

Martin M Brown (Chair), professor of stroke medicine, Institute of Neurology, University College London, UK. Thomas Liebig, professor of neuroradiology, department of Radiology, Uniklinik Köln, Germany; Theo Stijnen, professor of medical statistics, department of Medical Statistics and Bioinformatics at Leiden University Medical Center, The Netherlands.

### Trial managers

Esther S van der Heijden; Erasmus MC Rotterdam. Nadine M Fleitour, AMC Amsterdam.

## Electronic supplementary material

Additional file 1: **Statistical analysis plan.** (DOCX 118 KB)

## Abbreviations

ASPECTS: Alberta Stroke Program Early CT Score
CT: computed tomography
CTA: computed tomography angiography
DMC: data monitoring committee
DSA: digital subtraction angiography
IAT: intra-arterial treatment
IMS: Interventional Management of Stroke
IV: intravenous
MR CLEAN: Multicenter Randomized Clinical trial of Endovascular treatment for Acute ischemic stroke in the Netherlands
MRA: magnetic resonance angiography
MRI: magnetic resonance imaging
mRS: modified Rankin Scale
NIHSS: National Institutes of Health stroke scale
PROACT: Prolyse in Acute Cerebral Thromboembolism.
