# Supplementary material for: MR CLEAN, a multicenter randomized clinical trial of endovascular treatment for acute ischemic stroke in the Netherlands: study protocol for a randomized controlled trial
Source: Trials. 2014 Sep 1;15:343. doi: 10.1186/1745-6215-15-343 (PMC4162915; doi:10.1186/1745-6215-15-343)
Supplement: Supplementary file 1 — Additional file 1: Statistical analysis plan. (DOCX 118 KB) [file 13063_2014_2217_MOESM1_ESM.docx]

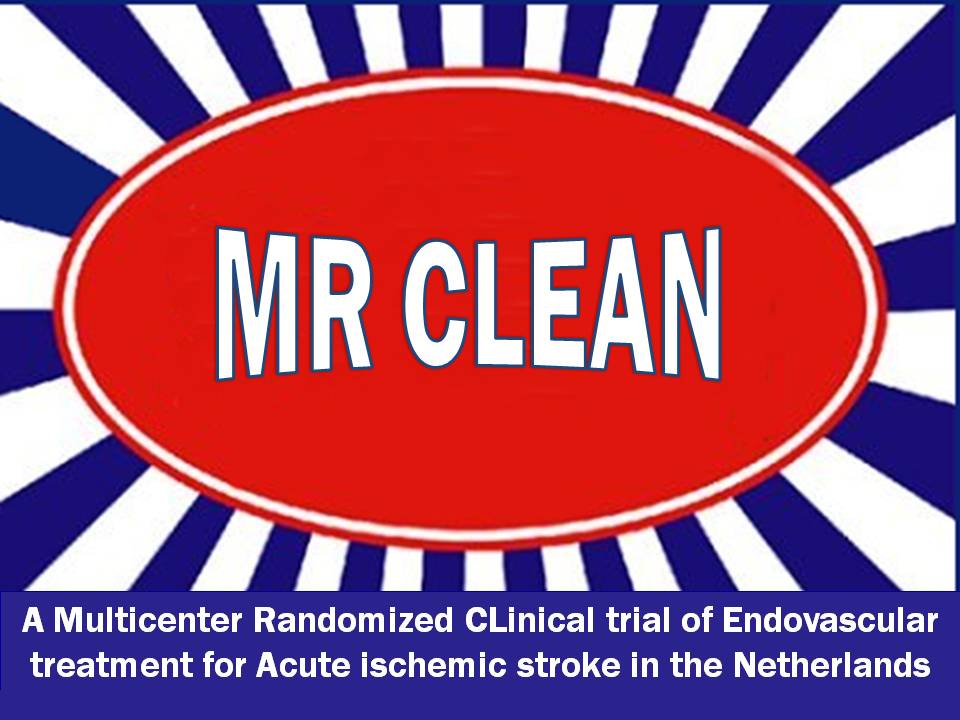
MR CLEAN SAP (statistical analysis plan).

Diederik Dippel, Hester Lingsma, Olvert Berkhemer.

for the MR CLEAN steering committee.

V 3.1 July 14, 2014.

# Introduction

The purpose of the Multi Center Randomized Clinical trial of Endovascular treatment in The Netherlands (MR CLEAN) is to assess the safety and effect on functional outcome of intra-arterial treatment in patients with acute ischemic stroke caused by intracranial arterial, anterior circulation occlusion.

Here we will summarize the statistical analysis of the data. We will describe how missing data will be handled and subgroup analyses will be performed. Moreover, we will describe the time path of the analyses and the process of deblinding, as well as reporting to the Data Monitoring and Safety Committee (DMSC).

Although we list an extensive number of analyses, we do not imply that all these will be described in the main paper, because of space restrictions.

# Status of the trial

The trial is running well. Inclusion has ended on March 16, 2014. In total, 502 patients have been randomized, but 2 patients have withdrawn consent, leaving 500 patients who have been registered in the database.

# Research questions

## Primary research questions

The primary objective of this study is to estimate the effect of endovascular treatment on functional outcome after acute ischemic anterior circulation stroke of less than six hours duration, in patients with a symptomatic intracranial occlusion.

## Secondary research questions

The secondary objectives are to assess the safety of endovascular treatment with regard to the occurrence of hemorrhagic and ischemic complications, the efficacy with regard to obtaining recanalization, and to evaluate predictors of recanalization, including imaging aspects and hemostatic parameters. Moreover, we want to assess the safety and efficacy of different types of endovascular treatment (i.e. mechanical treatment, intra-arterial thrombolysis) different combinations of treatment (i.e. with intravenous alteplase) and different timings of treatment.

# Trial design

This is a multicenter clinical trial with randomized treatment allocation, open label treatment and blinded endpoint evaluation (PROBE design).

The intervention contrast is intra-arterial treatment (alteplase or urokinase, and/or mechanical treatment) versus no intra-arterial treatment. The treatment is provided in addition to best medical management, which may include intravenous alteplase.

Randomization was stratified for center, dichotomized score on the National Institutes of Health Stroke Scale (NIHSS),[1] treatment with iv alteplase and intended mechanical treatment.

## Inclusion and exclusion criteria

Inclusion criteria are:

- A clinical diagnosis of acute stroke, with a deficit on the NIH stroke scale of 2 points or more.
- CT or MRI scan ruling out intracranial hemorrhage.
- Intracranial arterial occlusion of the distal intracranial carotid artery or middle (M1/M2) or anterior (A1/A2) cerebral artery, demonstrated with CTA, MRA, DSA.
- The possibility to start treatment within 6 hours from onset.
- Informed consent given.
- Age 18 or over.

General exclusion criteria are:

- Arterial blood pressure > 185/110 mmHg.
- Blood glucose < 2.7 or > 22.2 mmol/L.
- Intravenous treatment with thrombolytic therapy in a dose exceeding 0.9 mg/kg alteplase or 90 mg.
- Intravenous treatment with thrombolytic therapy despite contra-indications, i.e. major surgery, gastrointestinal bleeding or urinary tract bleeding within the previous 2 weeks, or arterial puncture at a non-compressible site within the previous 7 days.
- Cerebral infarction in the distribution of the relevant occluded artery in the previous 6 weeks.

Specific exclusion Criteria for intended mechanical thrombectomy are:

- Laboratory evidence of coagulation abnormalities, i.e. platelet count <40 x 10^9^/L, APTT>50 sec or INR >3.0.

Specific exclusion criteria for intended intra-arterial thrombolysis are:

- History of intracerebral hemorrhage.
- Severe head injury (contusion) in the previous 4 weeks.
- Clinical or laboratory evidence of coagulation abnormalities, i.e. platelet count <90 x 10^9^/L, APTT>50 sec or INR >1.7.

## Primary and secondary outcomes

The primary outcome is the score on the modified Rankin Scale at 90 days. Secondary outcomes concern imaging parameters, clinical parameters and safety parameters.

Imaging parameters are:

- Vessel recanalization at 24 hours after treatment, assessed by CTA or MRA. The criteria for recanalization on CTA or MRA are based on the Arterial Occlusive Lesion (AOL) scale,[2] and the Clot Burden Score.[3]
- Infarct size assessed by CT on day 5-7, using standard methods, including manual tracing of the infarct perimeter and semiautomated pixel thresholding.[4, 5]

Clinical parameters are:

- Score on the National Institutes of Health Stroke Scale (NIHSS) at 24 hours.
- Score on the NIHSS at 1 week or at discharge.
- Score on the EQ5D at 90 days,[6]
- Barthel index at 90 days.[7]

The primary safety parameter was neurologic deterioration within 24 hours from inclusion in the study. Neurological deterioration was defined as any decline in NIHSS of more than 4 points. In these patients, urgent brain CT is mandatory. This serious adverse event was further classified as due to intracranial hemorrhage, ischemia or other (undetermined) cause.

## Blinding

It was not possible to view the treatment allocation before the patient was registered in the study database, nor was it possible to remove the patient from the study base after treatment assignment has become known. Both patient and treating physician were aware of the treatment assignment. Information on outcome at three months was assessed through standardized forms and procedures by a dedicated research nurse. Assessment of outcome on the modified Rankin scale was based on this information, by assessors who were blind to the treatment allocation. Results of neuroimaging were also assessed in a blinded manner. Information on treatment allocation and 90-day outcome was kept separate from the main study database. The steering committee members were kept unaware of the results of interim analyses of efficacy and safety. The trial statistician combined data on treatment allocation with the clinical data in order to report to the data monitoring and safety committee (DMSC) in a closed session.

# Statistical analysis proper

## Primary effect analysis

The main analysis of this trial consists of a single comparison between the trial treatment groups of the primary outcome after 90 days. The analysis is based on the intention-to-treat principle. The primary effect parameter takes the whole range of the modified Rankin scale (mRS) into account and is defined as the relative risk for improvement on the mRS estimated as an odds ratio with ordinal logistic regression.[8] Multivariable regression analysis will be used to adjust for chance imbalances in main prognostic variables between intervention and control group in the primary effect analysis, but also in all secondary analyses and subgroup analyses.[9] These key variables are:

- age,
- stroke severity (NIHSS) at baseline
- time to randomization
- previous stroke,
- atrial fibrillation,
- diabetes mellitus and
- carotid top occlusion versus no carotid top occlusion

## Primary effect analysis in subgroups

The effect of intervention on the modified Rankin scale will be analyzed in the following subgroups:

- Age 80 or over versus age less than 80
- NIHSS in tertiles (2-15, 16-19 and 20 or higher)
- Carotid top occlusion present versus no carotid top occlusion
- Time since onset to randomization 120 minutes or less versus more than 120 minutes.
- Extracranial >50% carotid stenosis or occlusion versus no >50% carotid stenosis or occlusion
- ASPECTS 0-4 / 5-7 / 8-10
- Thrombus length >7mm versus 7 mm or less.

Secondary effect parameters will be the improvement according to the classical dichotomizations of the modified Rankin scale, neurological deficit at 24 hours and 1 week measured with the NIHSS, quality of life at 90 days measured with EQ5D and vessel patency at 24 hours as well as infarct size at 5-7 days on CT or MRI.

1. mRS 0-1 versus 2-6 at 90 days
2. mRS 0-2 versus 3-6 at 90 days
3. mRS at 0-3 versus 4-6 at 90 days
4. Barthel index 19-20 versus <19 at 90 days
5. Score on the EQ5D at 90 days
6. Score on the NIHSS at 24 hours
7. Score on the NIHSS at 7 days or discharge
8. Vessel patency (clot burden score) on CTA at 24 hours
9. Infarct size (automated volume measurement) on CT at 5-7 days.

Effect parameter in these first four analyses will be the odds ratio, estimated with multiple logistic regression. The effect parameter on the fourth to sixth outcome (EQ5D and NIHSS) will be a regression parameter beta, estimated with multiple linear regression models. Outcome data may have to be log-transformed. The clot burden score will be dichotomized into 10 or less than 10 and the effect parameter will be the odds ratio, estimated with multiple logistic regression. The effect on infarct size will be estimated with a multiple linear regression model.

In all analyses, statistical uncertainty will be expressed by means of 95% confidence intervals. Although the size of this study does not allow for precise estimates of treatment effect in subgroups, we assess heterogeneity of effects, and analyze consistency of effects on secondary outcomes.

## Missing data and death

Patients who die within the study period will be assigned the worst score on all outcome measures and taken into the analysis. Proportions of missing values for all variables will be reported. Variables that will be used to adjust the primary and secondary effect analyses (age, NIHSS at baseline, time to randomization, previous stroke, atrial fibrillation, diabetes mellitus and site of intracranial occlusion are designated as key variables. Missing values for these variables (if any) will be analysed for randomness and imputed with standard methods.

# Time path of the analyses and locking of the database.

To maximize time for analysis and interpretation of the results and allow presentation of the final results at the World Stroke Conference by the end of October 2014, a soft-lock and preliminary analysis will be performed once the last patients have had their final outcome recorded and the data has been reviewed by the DMSC by end of July 2014. Following final data cleaning on the last patients to be recruited, a hard-lock will be performed by mid September. The results of this analysis will be considered by the Trial Steering Committee by the end of September, 2014. The preliminary interpretation will be performed after soft-lock by DD, CM and HL; they will not be involved in resolving any final queries to maintain the integrity and blinding of the final database. The approach of soft-lock then hard-lock is a standard approach in large trials and allows more time to be spent on considering the results of a trial, their interpretation and presentation for publication.

# References

1. Brott T, Adams HP, Jr., Olinger CP, Marler JR, Barsan WG, Biller J, Spilker J, Holleran R, Eberle R, Hertzberg V *et al*: **Measurements of acute cerebral infarction: a clinical examination scale**. *Stroke* 1989, **20**(7):864-870.

2. Higashida RT, Furlan AJ: **Trial Design and Reporting Standards for Intra-Arterial Cerebral Thrombolysis for Acute Ischemic Stroke**. *Stroke* 2003, **34**(8):e109-e137.

3. Puetz V, Dzialowski I, Hill MD, Subramaniam S, Sylaja PN, Krol A, O'Reilly C, Hudon ME, Hu WY, Coutts SB *et al*: **Intracranial thrombus extent predicts clinical outcome, final infarct size and hemorrhagic transformation in ischemic stroke: the clot burden score**. *International journal of stroke : official journal of the International Stroke Society* 2008, **3**(4):230-236.

4. van der Worp HB, Claus SP, Bar PR, Ramos LM, Algra A, van GJ, Kappelle LJ: **Reproducibility of measurements of cerebral infarct volume on CT scans**. *Stroke* 2001, **32**(2):424-430.

5. Gavin CM, Smith CJ, Emsley HC, Hughes DG, Turnbull IW, Vail A, Tyrrell PJ: **Reliability of a semi-automated technique of cerebral infarct volume measurement with CT**. *CerebrovascDis* 2004, **18**(3):220-226.

6. EuroQol Group T: **EuroQol-a new facility for the measurement of health-related quality of life.** *Health Policy* 1990, **16**(3):199-208.

7. Mahoney FI, Barthel DW: **Functional Evaluation: The Barthel Index**. *Md State Med J* 1965, **14**:61-65.

8. McHugh GS, Butcher I, Steyerberg EW, Marmarou A, Lu J, Lingsma HF, Weir J, Maas AI, Murray GD: **A simulation study evaluating approaches to the analysis of ordinal outcome data in randomized controlled trials in traumatic brain injury: results from the IMPACT Project**. *Clin Trials* 2010, **7**(1):44-57.

9. Hernandez AV, Steyerberg EW, Butcher I, Mushkudiani N, Taylor GS, Murray GD, Marmarou A, Choi SC, Lu J, Habbema JD *et al*: **Adjustment for strong predictors of outcome in traumatic brain injury trials: 25% reduction in sample size requirements in the IMPACT study**. *J Neurotrauma* 2006, **23**(9):1295-1303.
